# AI-enabled label free monitoring of TGFβ1-induced remodeling in iPSC-derived alveolar organoids

**DOI:** 10.1016/j.mtbio.2026.103181

**Published:** 2026-05-07

**Authors:** Seung Hyeon Kim, Jooyeon Lee, Jong Hyeok Han, Sang Hyuk Seo, Jimin Jang, Jong-Hee Lee, Seok-Ho Hong, Myeong Jin Ju, Dae-Hee Lee, Hee-Jae Jeon, Se-Ran Yang

**Affiliations:** a Department of Mechanical and Biomedical Engineering, Kangwon National University, Chuncheon, 24341, Republic of Korea; b Department of Thoracic and Cardiovascular Surgery, School of Medicine, Kangwon National University, Chuncheon, 24341, Republic of Korea; c Department of Smart Health Science and Technology, Kangwon National University, Chuncheon, 24341, Republic of Korea; d National Primate Research Center (NPRC), Korea Research Institute of Bioscience and Biotechnology (KRIBB), Cheongju, Republic of Korea; e Department of Internal Medicine, School of Medicine, Kangwon National University, Chuncheon, Republic of Korea; f The University of British Columbia, Faculty of Medicine and Applied Science, Department of Ophthalmology and Visual Sciences, Vancouver, BC, V5Z 0A6, Canada; g Department of Marine Convergence Science, Kangwon National University, Gangneung, Gangwon State, 25457, Republic of Korea

**Keywords:** Pulmonary fibrosis, iPSC-derived alveolar organoids, Deep neural networks, Label-free longitudinal imaging

## Abstract

Pulmonary fibrosis (PF) is a progressive interstitial lung disease with poor prognosis and limited therapeutic options, largely due to the lack of physiologically relevant and dynamically traceable preclinical models. Here, we establish an induced pluripotent stem cell (iPSC)-derived alveolar organoid platform that faithfully recapitulates PF-like remodeling upon TGFβ1 stimulation. The organoids exhibit lineage commitment to the distal lung epithelium (NKX2.1, SFTPC/SFTPB) and, following TGFβ1 exposure, undergo hallmark fibrotic changes including organoid condensation and size reduction, collagen deposition (Masson's trichrome), myofibroblast activation (α-SMA), up-regulation of profibrotic genes (COL1A1, FN, VIM, ACTA2), and partial EMT-like reprogramming (↑CDH2, TWIST1, and ↓CDH1). To enable label-free, longitudinal readouts, we integrate a deep neural network (YOLOv8-nano) that detects subtle morphological cues directly from bright-field images and classifies treatment status with high fidelity. Across augmented datasets (8892 images), the model achieved strong performance on the original context-preserving images (mAP50–95 up to 0.95; high precision/recall and 98–99% true-positive rates), supporting robust discrimination of control versus TGFβ1-treated organoids. This AI-enhanced organoid system provides a quantitative, label-free platform for monitoring fibrotic remodeling and offers a scalable foundation for preclinical antifibrotic screening and mechanism-of-action studies.

## Graphical abstract

**Figure:**
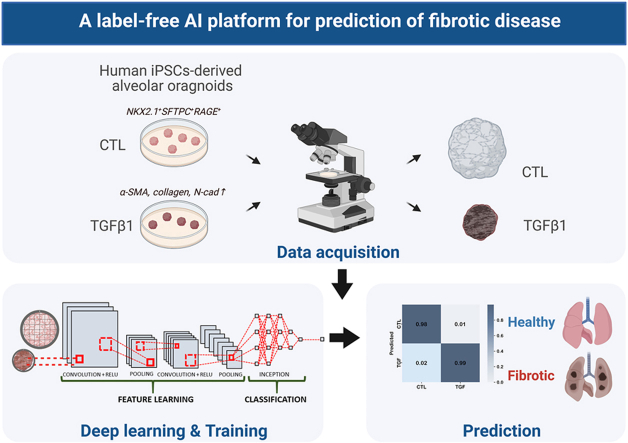

## 1 Introduction

Pulmonary fibrosis (PF) is a severe respiratory disease characterized by the progressive and irreversible deterioration of lung function, primarily due to disrupted interactions between cellular components and the extracellular matrix [1,2]. It is mainly characterized by excessive extracellular matrix accumulation, increased collagen deposition [3], extensive tissue remodeling, myofibroblast proliferation, and epithelial-mesenchymal transition (EMT) [4], making it challenging to replicate these complex biological processes *in vitro* [3,5]. The complexity of PF involves genetic predispositions and environmental factors, such as exposure to harmful substances and autoimmune reactions, complicating the development of accurate models and targeted treatments [6].

To address these challenges, advanced models are crucial in the discovery of effective treatments [[6], [7], [8]]. Alveolar structures, primarily composed of Type I (AEC1) and Type II (AEC2) alveolar epithelial cells, are central to the study of PF [9,10]. AEC1 cells form fragile barriers essential for gas exchange. Whereas AEC2 cells not only synthesize pulmonary surfactants but also possess self-renewal capacity and serve as facultative progenitors that proliferate and differentiate into AEC1 after injury, thereby supporting alveolar regeneration and homeostasis [11]. Leveraging cutting-edge biotechnological advancements, we used induced pluripotent stem cells (iPSCs) that differentiate into alveolar epithelial cells [12].

Recent advancements in organoid technology have enabled the development of more sophisticated *in vitro* models that closely mimic the cellular architecture and physiological responses of human tissues, including the lungs [13]. These organoid models are valuable for disease modeling, drug testing, and personalized medicine [14,15]. However, traditional organoid analysis methods often involve invasive procedures, such as cellular staining, which can disrupt the functional state morphological architecture of the organoids [16,17]. Additionally, these methods typically provide only static information, limiting their effectiveness in dynamic, label-free, longitudinal analysis [18,19]. The static nature of these analyses fails to capture the ongoing progressive changes in PF, highlighting the need for methods that offer continuous insights into cellular and molecular dynamics [18,20].

**Fig. 1:**
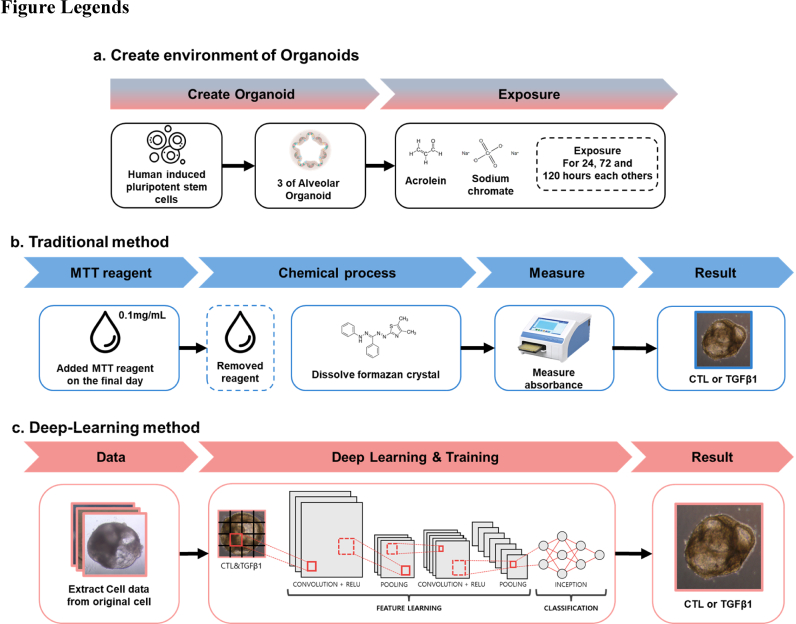
Schematic diagram of workflow for alveolar organoid analysis with deep learning and MTT method. (a) iPSC-derived alveolar organoid generation and differentiation of alveolar progenitor cells. For induction of fibrotic remodeling, organoids were exposed to TGFβ1 for the indicated durations (24–120 h). (b) Viability assessment via MTT assay, spectrophotometric quantification, and image collection. (c) Deep-learning pipeline for label-free analysis: image acquisition, preprocessing, network inference, and computational readout.

Advanced imaging and computational modeling provide insight into cell–matrix interactions, enabling quantitative analysis of disease mechanisms across molecular to cellular scales. To address the limitations of conventional 3-(4,5-dimethylthiazol-2-yl)-2,5-diphenyltetrazolium bromide (MTT) assays and other invasive techniques, we employed advanced artificial intelligence (AI) to analyze organoid images under both treated and untreated conditions, as illustrated in Fig. 1. Our AI-driven platform used deep learning algorithms trained on a comprehensive dataset of high-resolution organoid images, enabling the identification and categorization of subtle morphological alterations indicative of fibrosis progression. The AI application allowed for non-invasive monitoring of the fibrotic status and viability of organoids by recognizing visible cues and patterns corresponding to various stages of disease progression, thereby preserving their structural and cellular integrity. This approach reduces reliance on destructive staining and endpoint assays, facilitating nondestructive, label-free, longitudinal monitoring of cellular responses to treatments. Consequently, our approach enhances experimental efficacy and precision while significantly reducing time and resources required for data acquisition and analysis.

Integrating AI with advanced organoid models represents a significant advancement in regenerative medicine, offering a dynamic, label-free, longitudinal perspective on disease processes. This approach deepens our understanding of PF at the granular level and opens new avenues for AI applications in biomedical research. By transitioning from traditional static methods to dynamic, AI-enhanced methodologies, this study revolutionizes the modeling, monitoring, and potential treatment of complex disorders such as PF.

## 2 Methods

### 2.1 Recombinant protein and reagents

Activin A (338-AC), BMP4 (314-BP), FGF10 (345-FG), KGF (251-KG), and Noggin (6057-NG) were purchased from R&D Systems (Minneapolis, MN, USA). CHIR99021 (1386) and SB431542 (1614) cells were acquired from Axon (AZ, USA). ATRA (R2625), 8-Br-cAMP (B5386), dexamethasone (D4902), IBMX (I5879), L-ascorbic acid (A4544), and sodium butyrate (CHB5587) were sourced from Sigma-Aldrich (Sigma, MO, USA). ITS (41400045) and B27 (17504044) were purchased from Gibco (Grand Island, NY, USA).

### 2.2 hiPSC cell culture

Human iPSCs (C3) were obtained from the Korea Research Institute of Bioscience and Biotechnology (KRIBB, Daejeon, Korea) and cultured using previously described methods [21]. In brief, cells were cultured in TeSR™E8™ basal medium (STEMCELL Technologies, Vancouver, Canada) on dishes coated with vitronectin (STEMCELL Technologies, Vancouver, Canada). For subculture, colonies were mechanically fragmented using the 10 μL tips, and transferred to fresh vitronectin-coated dishes with basal medium containing Y-27632 (Tocris, Bristol, UK).

### 2.3 Differentiation of hiPSCs into alveolar epithelial cells (iAECs)

hiPSCs were differentiated into alveolar epithelial cells according to the protocol described in our previous study [21]. Briefly, 10 to 20 small colony fragments were seeded on vitronectin-coated 6 well plates with Y-27632. Once the colonies reached an approximate diameter of 500 μm, the culture medium was replaced with differentiation medium. Subsequently, the medium was freshly replaced daily with appropriate medium for each corresponding day. The differentiation media for each corresponding day are shown in Table 1.

**Table 1:** The components used in the differentiation medium for AECs.

Day	D0	D1	D2-D5	D6-D9	D10-D13	D14 (Forced aggregation)	D15-D20	D21-D25
**Supplements**	100 ng/mL Activin A			0.05 mg/mL L-ascorbic acid				0.25% BSA
	1uM CHIR99021			0.4 mM monothioglycerol				0.1 % ITS
	2 × B27							1 × B27
	-	0.25 mM sodium butyrate	0.125 mM sodium butyrate	100 ng/mL noggin	20 ng/mL BMP4	10 ng/mL FGF10		50 nM dexamethasone
				10 μM SB431542	0.5 μM ATRA	3 μM CHIR99021		0.1 mM IBMX
					3.5 μM CHIR99021	10 ng/mL KGF		100 ng/mL KGF
						20 μM DAPT		0.1 mM 8-br-cAMP
**Basal media**	RPMI-1640 (1% p/s)			DMEM/F12 (1% p/s)				DMEM Ham's F12 (1% p/s)
**Information of Basal media**	RPMI-1640			A1049101 (Gibco)				
	DMEM/F12			11330-032 (Gibco)				
	DMEM Ham's F12:			D8437 (Sigma)				

### 2.4 Generation of AOs and fibrosis organoids

AOs were generated as described previously [21]. Briefly, cells were detached on D14 of iAEC differentiation with 0.4 unit/mL collagenase B for 20 min and dissociated with TrypLE™ Express Enzyme (Gibco, MD, USA) for 10 min in 37 °C. The cells were seeded into Corning® 96-well Round Bottom Ultra-Low Attachment Microplate (Corning, USA) at a seeding density of 7 × 10^3^ cells/well in 100 μL. For forced aggregation of cells, the plate was centrifuged for 4min at 1500 rpm. 50 μL of Matrigel solution, diluted 1:7 in basal medium, was carefully added onto aggregated cells. After overnight culture at 37 °C, the aggregated cells were transferred into Corning® ultra-low attachment plates with freshly prepared differentiation medium and cultured until D25. To induce fibrosis organoid, AOs were treated with 20 ng/mL TGFβ1 on D21 for 3 days.

### 2.5 Quantitative label-free, longitudinal PCR analysis

Total RNA was isolated from AOs using the AccuPrep® Universal RNA Extraction Kit (Bioneer, Korea), followed by cDNA synthesis with the Reverse Transcription Master Premix (Elpis Biotech, Korea). PCR amplification was performed with 10 pmol of specific primers, 10 ng cDNA, and TOPreal™ qPCR 2X PreMIX (SYBR Green) (Enzynomics, Korea) on a Step One Plus label-free, longitudinal PCR system (Applied Biosystems, Warrington, UK). Relative expression was calculated by the 2-ΔΔCt method, with GAPDH serving as an internal control. Primer sequences are listed in Table 2.

**Table 2:** Sequences of primers used in label-free, longitudinal PCR.

Genes	Sequence
SOX2	F: 5′-GCAAGATGGCCCAGGAGAA-3′
	R: 5′-CCAGGCGCTTGCTGAT-3′
SOX9	F: 5′-GACGTCATCTCCAACATCGA-3′
	R: 5′-GTTGGGCGGCAGGTACT-3′
GATA6	F: 5′-CGGGAGAGAGCACCAATC-3′
	R: 5′-GCCCATCTTGACCCGAAT-3′
NKX2.1	F: 5′-CCGTTCTCAGTGTCTGACATC-3′
	R: 5′-CCTCCATGCCCACTTTCTT-3′
SFTPC	F: 5′-GAGTCCACCGGATTACTCG-3′
	R: 5′-GATGAGAAGGCGTTTGAGG-3′
SFTPB	F: 5′-GAGCCGATGACCTATGCCAAG-3′
	R: 5′-AGCAGCTTCAAGGGGAGGA-3′
COL1A1	F: 5′-AGTGGGAGACCTCGAGAAGA-3′
	R: 5′-TTGGTCGGTGGGTGACTCTG-3′
FN	F: 5′-GAGCCGATGACCTATGCCAAG-3′
	R: 5′-ACTGTGACAGCAGGGAGCA-3′
VIM	F: 5′-ACACCCTGCAATCTTTCAGAGAC-3′
	R: 5′-GATTCCACTTTGCGTTCAAGGT-3′
ACTA2	F: 5′-TGCCTGATGGGCAAGTGA-3′
	R: 5′-CTGGGCAGCGGAAACG-3′
CDH1	F: 5′-AAGGAGGCGGAGAAGAGGAC-3′
	R: 5′-CGTCGTTACGAGTCACTTCAGG-3′
HOPX	TCAACAAGGTCGACAAGCAC
	TCTGTGACGGATCTGCACTC
AGER	GCCACTGGTGCTGAAGTGTA
	TGGTCTCCTTTCCATTCCTG
TWIST1	CCACTGAAAGGAAAGGCATC
	AGGCCAGTTTGATCCCAGTA
CDH2	GACAATGCCCCTCAAGTGTT
	CCATTAAGCCGAGTGATGGT
GAPDH	F: 5′-CAATGACCCCTTCATTGACC-3′
	R: 5′-GACAAGCTTCCCGTTCTCAG-3′

### 2.6 Histological analysis

AOs were collected, and conditioned medium was eliminated in a 15 mL tube. 4% paraformaldehyde was added onto AOs to fix them. After washing twice with 0.1% BSA solution, AOs were placed from a tube into the Disposable plastic Cryomold™ molds (Cat. No. 4565, Sakura Finetek, Japan). 60 °C Heated Epredia™Histogel™Specimen Processing Gel was poured into the molds, and immediately relocated onto ice for 10 min. Samples were embedded in paraffin and sectioned at 6 μm. Hematoxylin and eosin staining was performed to observe the cross sections of AOs.

### 2.7 Immunofluorescence staining

Paraffin-embedded sections were incubated at 60 °C for 20 min to solidify, followed by deparaffinization using xylene (twice for 10 min each). Rehydration was achieved through a graded ethanol series (100% to 70% EtOH, 5 min per step). For antigen retrieval, slides were boiled in citrate buffer using an autoclave (121 °C for 1 min). For blocking, slides were treated with 10% normal goat serum for 1 h. The slides were then incubated with primary antibody (diluted 1:500 in blocking buffer) overnight, followed by incubation with secondary antibodies (diluted 1:1000 in PBS) for 1 h. Antibody details are as follows: AQP5, sc-514022, Santa Cruz; SFTPC, ab-90716, Abcam; RAGE, sc-365154, Santa Cruz; EpCAM, #93790, Cell Signaling Alexa 594, goat anti-rabbit, A-11012; Alexa 488, goat anti-mouse, A11001, Invitrogen. After three washes, DAPI solution (diluted 1:1000 in PBS, Invitrogen, USA) was applied to the AO sections for 1 min and then covered with a slide coverslip. Confocal microscopy (LSM880 with Airyscan, Carl Zeiss, Germany; FV3000, Olympus, Japan) was used for observation.

### 2.8 Statistical analysis and AO image acquisition

The results are expressed as mean ± standard error of the mean. One-way analysis of variance followed by Bonferroni's multiple comparison test was performed to compare multiple groups. For comparisons between two experimental samples, an unpaired two-tailed Student's t-test was performed. Statistical significance was set at p < 0.05. Also, to acquire AO image, on day 25 of differentiation, AOs were visualized using an optical microscope (CKX41; Olympus, Tokyo, Japan). Images were captured at 40 × magnification. A total of 230 images were captured, consisting of 168 controls and 62 TGFβ1. The images are shown in Supplementary Fig. S1. All experiments were independently repeated at least three times to ensure reproducibility. Each experiment included multiple biological replicates derived from different organoid batches.

### 2.9 Datasets

**Fig. 2:**
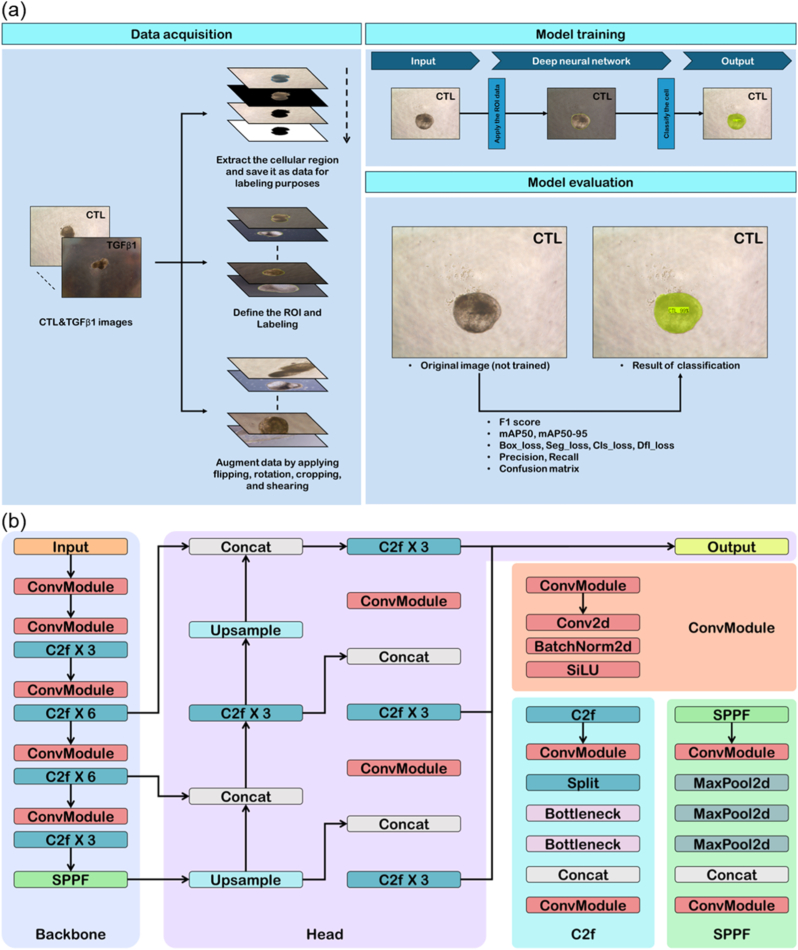
**Process flow for training a deep neural network for image classification of alveolar organoid.** (a) Data Acquisition step involves the collection of cellular images labeled as ‘Control’ (CTL) and treated with TGFβ1. (i)The images undergo processing to extract the cellular region, which is saved as data for labeling purposes. Regions of Interest (ROIs) are defined, and the images are labeled accordingly. Subsequently, data augmentation is performed through various techniques like flipping, rotation, cropping, and shearing to enhance the dataset. The prepared and augmented images serve as inputs to the deep neural network for the model training step. (ii) This figure depicts the flow from inputting the cellular image labeled CTL, applying image of ROIs, to the model classifying the cell and highlighting the identified region in the output image. The evaluation stage showcases a comparison between the original, untrained image and the result of classification post-training. The image on the left represents the cellular image before model training, while the image on the right shows the successful identification and classification of the cell as CTL, highlighted in green overlay. (iii) The model's performance is quantitatively assessed using metrics such as F1 score, mAP50, precision, recall, several loss functions (losses of box, segmentation, class, distribution focus), and the confusion matrix. (b) Structure of YOLOv8. Backbone extract features from input images, such as shapes, colors, and textures. Head predict the classes and location of object and improves the accuracy of detecting object to create final output. These features enable the model to effectively detect and classify objects within the images. Head uses features that extracted by the Backbone to detect accurately the position of objects and classify them. ConvModule: Convolution Module, C2fX3, C2fX6: CSP Bottleneck with 2 convolutions, SPPF: Spatial Pyramid Pooling-Fast, Conv2d: 2-Dimensional Convolutional Layer, BatchNorm2D: 2-Dimensional Batch Normalization Layer, SiLU: Sigmoid Linear Unit. (For interpretation of the references to color in this figure legend, the reader is referred to the Web version of this article.)

We created three types of datasets: the first comprised only images with a background, the second consisted of images without a background, and the third combined the first two datasets. Initially, images with backgrounds were commonly used in deep learning. We investigated the impact of the background on model training between datasets with and without a background. We trained the model using three datasets and calculated the results with convolution neural network. Object detection is a significant research area in computer vision, it has been widely applied to various fields [22,23]. Over the years, deep learning-based object detection has remarkable development, many algorithms like YOLO have shown continuously. YOLOv8 is basically same as that of YOLOv5 and used C2f modules, it obtained more abundant gradient flow information while ensuring its light weight [24]. SPPF modules connected three maxpools to guarantee the accuracy of objects in various scales while ensuring a light weight simultaneously. Architecture consists of Backbone and head. The backbone is a pre-trained convolutional neural network that extracts low, medium, and high-level feature maps from an input image, it passes them onto the head, classifying objects and predicting bounding boxes. The convolution neural network that used in these experiments is YOLOv8-nano, the smallest parameters of YOLOv8's five variants' parameters (Fig. 2). To assess and control potential background confounding, we compared training on (i) original context-preserving images, (ii) background-removed images, and (iii) a combined dataset. Preprocessing included intensity normalization and ROI-focused cropping to bias learning toward organoid morphology rather than plate- or illumination-specific cues.

### 2.10 Training environment

The optimizer used was AdamW, an adaptive gradient algorithm. AdamW performs better than Adam at learning late schedules and provides a more separable hyperparameter search space [25] [24]. The loss functions were varifocal loss, distribution focal loss, and complete intersection-over-union loss (CIOU). Varifocal loss (VFL), inspired by focal loss, is a loss function for training a dense object detector to predict the IACS. Unlike focal loss, which treats positives and negatives equally, VFL treats them asymmetrically. Distribution focal loss was used to address the class imbalance in datasets with very rare objects, ensuring the model's efficient detection of these rare objects. CIOU is an enhancement over the traditional intersection-over-union loss. CIOU considers the overlap between the predicted and ground-truth boxes, the distance between the box centers, and the aspect ratio, providing a more comprehensive measure of box alignment [26]. We selected a learning rate and batch size of 0.001 and 64, respectively. The dataset that used in the experiment consisted of 8892 images, with 4401 from control and 4491 from TGFβ1 organoids in Table 3. These 8892 training images were generated from 230 original AO images through data augmentation techniques, including flipping, rotation, cropping, and shearing, as described in Section 2.11. To minimize class bias, we used class-aware sampling to form ∼1:1 balanced mini-batches and applied class-weighted classification terms in the loss.”

**Table 3:** Basic information of deep-learning options and dataset.

Parameters	
Architecture	YOLOv8
Optimizer	AdamW
Loss function	VFL Loss, DFL Loss & CIOU Loss
Learning rate	1E-3
Batch size	64
Total number of epochs run during training	600

Dataset	Number of cells	Number of images
Training	8379	8379 (3363 normal, 5016 impaired)
Validation	342	342 (142 normal, 200 impaired)
Final test	171	171(72 normal, 99 impaired)

### 2.11 Data acquisition and augmentation

Initially, a diverse set of cellular images was collected, capturing various states of AOs under control conditions and after exposure to TGFβ1. These images underwent a meticulous preprocessing routine to isolate and define ROIs, which are crucial for accurate model training in Supplementary Figs. S2-S4. Each image was labeled appropriately to reflect its condition CTL or TGFβ1. To enhance the robustness of our dataset, we employed a series of data augmentation techniques, including flipping, rotation, cropping, and shearing. These steps are vital for increasing the variability and volume of training data, thus preventing overfitting and improving the model's ability to generalize across new, unseen images. Augmentation was applied in a class-aware manner to improve minority representation and support balanced batches during training.

### 2.12 Model training and classification performance

To mitigate data leakage and overfitting and to ensure reliable generalization, images from the same organoid and imaging session were not split across training, validation, and test sets. All summary metrics are reported on a held-out test set without plate/session overlap with the training data. The prepared dataset was fed into a DNN trained to classify images based on the defined ROIs. The meticulously documented training process demonstrated the transformation from raw cellular images to a trained model capable of precise classification [27,28]. After training, the model demonstrated a significant aptitude for accurately identifying and classifying cellular images. For example, in the evaluation stage, a comparative analysis between the pre- and post-trained images illustrated the model's enhanced capability to identify control AOs, with the treated cells distinctly highlighted with a green overlay to signify successful classification (Fig. 2b and Supplementary Fig. S4).

### 2.13 Flow cytometry

To assess the expression of SFTPC and RAGE proteins, alveolar organoids were dissociated into single cells using TrypLE Reagents (Gibco, USA). The cells were collected in PBS containing 10% fetal bovine serum (FBS). For detection of the membrane protein RAGE, cells were first incubated with an anti-RAGE antibody (Santa Cruz Biotechnology, sc-365154) for 30 min, followed by staining with an Alexa Fluor 488–conjugated secondary antibody for 20 min. To detect intracellular SFTPC, cells were fixed and permeabilized using the BD Cytofix/Cytoperm™ Fixation/Permeabilization Kit (BD Biosciences, USA) according to the manufacturer's instructions. Cells were then incubated with an anti-SFTPC antibody (Abcam, ab312850) for 30 min, followed by staining with an Alexa Fluor 647–conjugated secondary antibody for 20 min. Cells were analyzed using BD Accuri™ C6 Flow Cytometer (BD Biosciences, USA). For the secondary antibody-only control, cells were processed identically, without incubation with primary antibodies and with secondary antibodies only.

### 2.14 Quantitative performance metrics

The efficacy of the model was quantitatively assessed using several performance metrics including the F1 score, mean average precision (mAP50), precision, and recall. Additionally, various loss functions, such as box loss, seg loss, cls loss, and dif loss, were monitored to further refine the training process [29]. A confusion matrix was utilized to provide a clear visual depiction of the model's performance, indicating a high level of accuracy in distinguishing between control AOs and TGFβ1-treated organoids.

## 3 Results

### 3.1 Generation NKX2.1^+^SFTPC ^+^ alveolar organoids (AOs) from hiPSCs

**Fig. 3:**
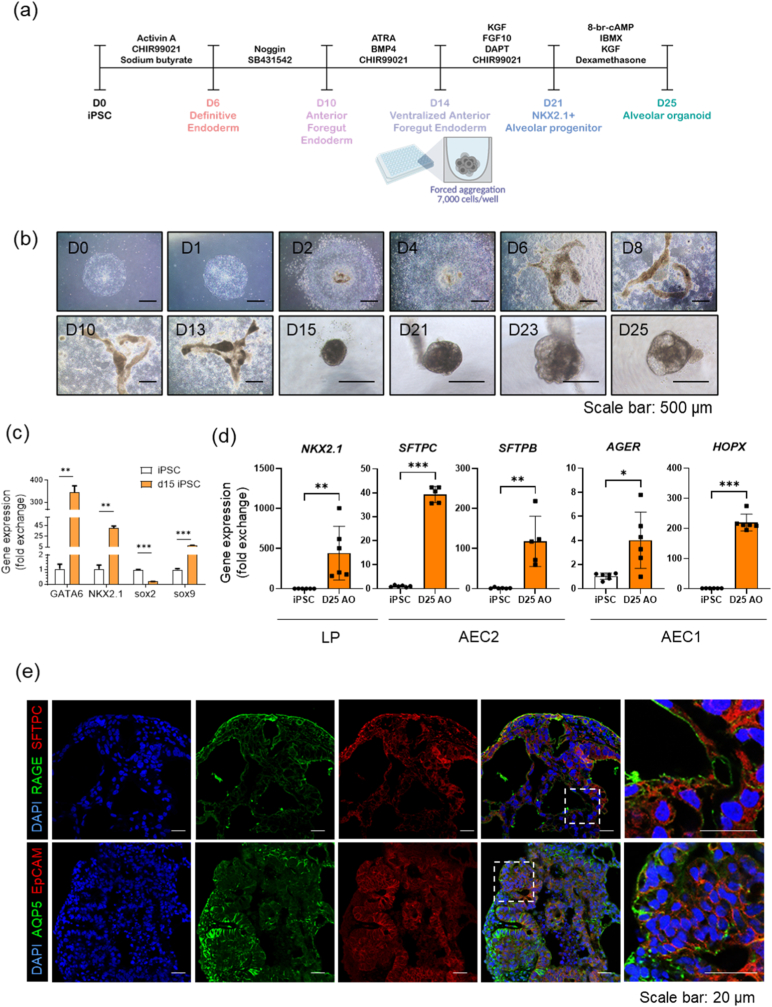
**Developmental progression and molecular characterization of alveolar organoids derived from hiPSCs.** (a) A representation of the initial culture and differentiation protocol for hiPSCs over a span of 25 days as outlined in the methods section. (b) A visual timeline showing the morphological changes in iPSCs from Day 0 (colony-like morphology) to Day 14 (detachment from culture plates) and subsequent aggregation with an extracellular matrix protein mixture by Day 15. (c) An analysis of the expression levels of lung epithelial lineage markers GATA6, NKX2.1, and SOX9, and the decrease in pluripotency marker SOX2, on Day 15 of differentiation. (d) RT-PCR analysis showing increased expression of lung progenitor cells (LP) and alveolar epithelial cell maturation markers, including NKX2.1, SFTPC, SFTPB, AGER, and HOPX, from Day 21 to Day 25. This upregulation is consistent with the observed morphological maturation characterized by robust membrane formation and the appearance of distinct transparent spaces. (e) Immunostaining data revealing the expression of alveolar epithelial cell type 1 (AEC1) markers RAGE and AQP5, type 2 (AEC2) marker SFTPC, and epithelial cell marker EpCAM, confirming the successful differentiation and characterization of the iPSC-derived alveolar organoids.

To explore the potential use of hiPSCs in PF organoid models, we developed AOs using a 25-day differentiation protocol, as outlined **in** Fig. 3a. Initially, hiPSCs were maintained under culture conditions that preserved their colony-like morphology, crucial for retaining pluripotency before differentiation (Fig. 3b; D0). By Day 14, these progenitor cells were induced to differentiate into iAECs and dissociated thereafter (Fig. 3b; D14). Following dissociation, the cells aggregated within a specialized extracellular matrix protein mixture to mimic the pulmonary basal lamina microenvironment. The matrix was removed the following day to promote self-organization into spheroids (Fig. 3b; D15).

During this phase, the differentiating cells initiated the expression of markers, indicating lung epithelial lineage commitment. By Day 15, high expression levels of the transcription factors GATA6, NKX2.1, and SOX9 essential for lung development were observed [30], whereas the pluripotency marker SOX2 showed low expression levels (Fig. 3c). This molecular shift represents a pivotal point in the differentiation trajectory, as the expression of these markers is crucial for guiding the subsequent maturation of organoids into functional lung tissue.

From Days 21 to 25, the differentiating spheroids exhibited notable morphological changes. The organoids developed a robust membrane structure and began to exhibit distinct transparent spaces reminiscent of alveolar architecture, indicating maturing alveolar epithelial cells. Additionally, a slight increase in size was observed, suggesting cellular expansion and extracellular matrix remodeling within the organoid structures (Fig. 3b; D21-D25). By Day 25, the organoids maintained consistent expression of NKX2.1 and exhibited notable levels of AEC2 markers (SFTPC and SFTPB) as well as AEC1 markers (AGER and HOPX), which are characteristic of distal alveolar epithelial cells (Fig. 3d) [31]. Flow cytometry analysis showed that SFTPC- and RAGE-positive cells were more prominently detected in AOs compared with undifferentiated iPSCs. Specifically, 47.6% of cells were SFTPC^+^RAGE^−^, while 30.4% of cells were double positive for SFTPC and RAGE in AOs. Previous studies have suggested that SFTPC^+^RAGE^+^ double-positive cells may represent a transitional state during the differentiation from AEC2 to AEC1 [32]. In addition, 10.0% of cells in the organoids were SFTPC^−^RAGE^+^, corresponding to AEC1-like populations (Supplementary Fig. S5).

Furthermore, immunostaining performed on Day 25 validated the presence of the AEC1 markers RAGE and AQP5, along with the AEC2 marker SFTPC, and the pan-epithelial cell marker EpCAM. This expression profile supported the successful differentiation and functional maturation of hiPSC-derived AOs (Fig. 3e). Taken together, we successfully generated NKX2.1^+^SFTPC^+^alveolar organoids from iPSC that exhibit alveolar epithelium-like structures and express distal alveolar cell phenotypes.

### 3.2 Morphological and structural changes induced by TGFβ1

**Fig. 4:**
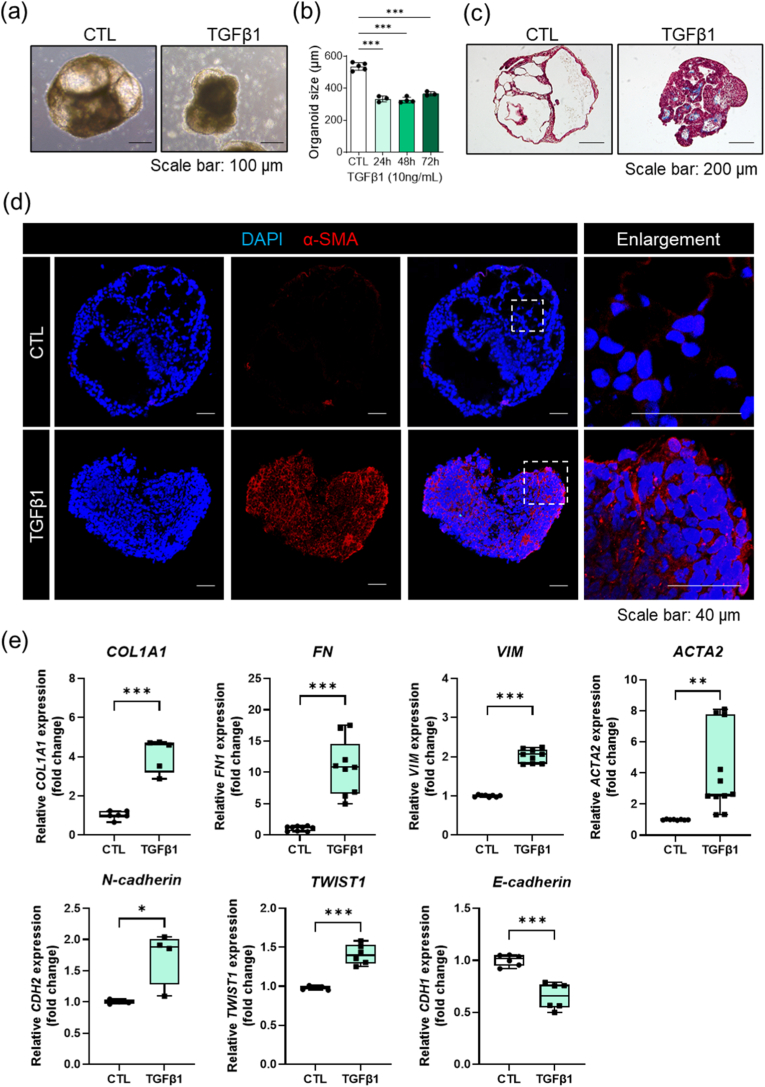
**Induction of pulmonary fibrosis in alveolar organoids via TGFβ1 treatment and subsequent molecular characterization.** (a) The figure depicts the changes in alveolar organoid morphology when exposed to TGFβ1, highlighting a reduction in size and structural differences when compared with the control. (b) A bar graph showing the significant decrease in the size of alveolar organoids post-TGFβ1 treatment as opposed to the control alveolar organoids. (c) Staining data illustrating collagen accumulation in TGFβ1-treated alveolar organoids in comparison to the control, indicating fibrotic development. The control alveolar organoids resemble alveolar sacs with thin epithelial linings, which are absent in TGFβ1-treated alveolar organoids. (d) Fluorescence microscopy images showing the expression of alpha-smooth muscle actin (α-SMA) as a myofibroblast marker, which is increased in TGFβ1-treated alveolar organoids, signifying fibrosis, whereas it is barely detectable in control alveolar organoids. (e) RT-PCR results showing the upregulation of pro-fibrotic genes (COL1A1, FN1, VIM, and ACTA2) in TGF-β1–treated alveolar organoids. The increased expression of CDH2 and TWIST1, along with the downregulation of the epithelial marker CDH1, indicates the occurrence of epithelial-to-mesenchymal transition (EMT).

We exploited the fibrogenic potential of transforming growth factor beta 1 (TGFβ1) to induce substantial alterations in the morphology of AOs derived from human induced pluripotent stem cells (hiPSCs), as shown **in** Fig. 4a. Treatment with TGFβ1 resulted in a progressive and significant reduction in organoid size over a period from 24 to 72 h, as shown in Fig. 4b. The treated organoids had a dense, condensed structure, contrasting with the expansive sac-like architecture of the untreated controls (Fig. 3a). High-resolution imaging revealed these transformations, highlighting the loss of the thin squamous epithelial lining typically observed in healthy alveolar structures. Throughout the treatment period, we observed an increase in density and loss of transparency within the organoids, indicative of the early-stage fibrotic transformation. These changes were meticulously documented using high-resolution imaging techniques that illustrated the physical condensation and alterations in extracellular matrix composition and cell–cell interactions.

### 3.3 Fibrotic remodeling and myofibroblast activation

Our histological assessments demonstrated a significant increase in collagen deposition in TGFβ1-treated AOs compared with that in healthy controls, indicating robust fibrotic remodeling. Collagen accumulation, a hallmark of PF, leads to the formation of dense fibrotic nodules that replace the normal alveolar parenchyma [19]. Masson's trichrome staining revealed substantial collagen accumulation in TGFβ1-treated organoids compared with controls, which retained the appearance of alveolar sacs with thin epithelial linings (Fig. 4c). Fibrotic transformation is distinguished by structural condensation and loss of typical alveolar spaces, reflecting extensive tissue remodeling [19].

In addition to histological observations, immunofluorescence analysis further validated these fibrotic alterations. As shown in Fig. 4d, fluorescence microscopy images revealed the expression of alpha-smooth muscle actin (α-SMA), a well-recognized myofibroblast marker [33]. In the treated organoids, α-SMA expression was significantly elevated, indicating active myofibroblast formation and fibrosis [9]. By contrast, α-SMA was slightly detectable in the control organoids, highlighting the specific induction of myofibroblast phenotypes following exposure to TGFβ1 [34]. The images clearly depicted the clear disparity in α-SMA expression between treated and untreated samples, highlighting the direct impact of TGFβ1 on cellular behavior and fibrotic pathway activation. These detailed visual and histological analyses confirmed that the structural and cellular changes induced by TGFβ1 are profound as well as critical for understanding the mechanisms influencing fibrosis in this *in vitro* model [35]. By documenting these changes using high-resolution imaging, we provided a clear visualization of the fibrotic processes, offering valuable insights into the cellular and molecular interaction underlying the progression of PF. This characterization supports the potential of our model as a robust platform for investigating the pathophysiology of fibrosis and testing new therapeutic approaches in controlled microenvironments.

### 3.4 Molecular indicators of pulmonary fibrosis-like remodeling in AOs

Our molecular assessments elucidated the cellular transformations induced by TGFβ1 in AOs. Label-free, longitudinal polymerase chain reaction (PCR) analysis revealed significant upregulation of fibrotic markers, including collagen type I alpha 1 chain (COL1A1), fibronectin (FN), vimentin (VIM), and α-smooth muscle actin (ACTA2), following TGFβ1 treatment [35]. This expression pattern indicates an active fibrotic remodeling process characterized by the replacement of normal tissue architecture with fibrotic tissue [36]. Concurrently, there was a notable upregulation of N-cadherin (CDH2) and TWIST1, accompanied by a downregulation of the epithelial marker E-cadherin (CDH1), indicating that TGFβ1 induces EMT, a key feature of fibrosis progression (Fig. 4e) [9,33,37].

Taken together, our findings demonstrated that TGFβ1 induces significant changes in gene expression characteristic of myofibroblast activation and fibrosis [35,38,39]. Fluorescence microscopy images and gene expression analysis provided effective visualization and quantification of these transformations, affirming the effectiveness of our *in vitro* model in replicating intricate disease mechanisms, such as those seen in PF.

### 3.5 Deep neural network development for AO image classification

We developed a DNN to classify images of AOs into “Control” (CTL) and TGFβ1-treated categories. As depicted in Fig. 2a, the data acquisition and preprocessing involved resizing the images from 2048 × 1536 to 640 × 480 pixels, extracting cellular isolation, defining regions of interest (ROI), and labeling each image. To address the limited size of the initial dataset, we applied data augmentation techniques, such as flipping (horizontal and vertical), rotating (45° to 135 °, both clockwise and counterclockwise), and distortion (horizontal and vertical range from −10 to +10). This expanded the dataset to 8892 images, comprising 4401 control and 4491 TGFβ1-treated images, ensuring a robust dataset for model training.

During training, the augmented dataset was used to train the DNN, with the ROI of each image input into the model for classification, as shown in Fig. 2b. Model performance was evaluated using various metrics, including the F1 score, mean average precision (mAP50, mAP50-95), precision, recall, and confusion matrix, along with loss functions such as box loss, segmentation loss, class loss, and distribution focus loss. These evaluations confirmed the model's high accuracy and reliability in detecting subtle morphological changes induced by TGFβ1 treatment and are critical for precise pathological assessment, thereby providing a valuable tool for label-free, longitudinal analysis of organoid models in biomedical research.

### 3.6 Enhanced AO analysis using deep learning: integrating image contextual and robust training for accurate fibrosis detection

**Fig. 5:**
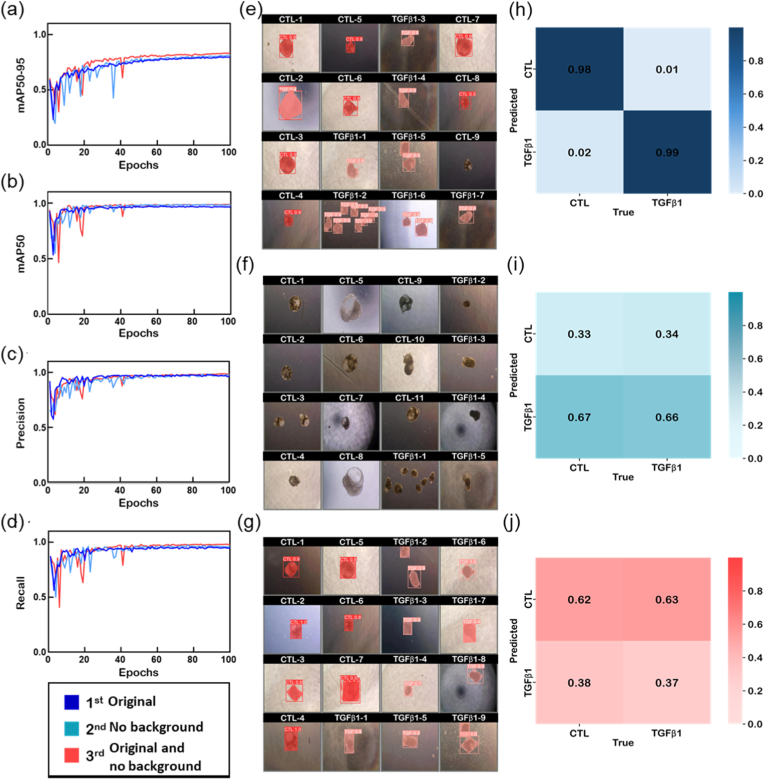
**Performance metrics and visual outcomes for cell classification via deep learning across multiple datasets.** (a)-(d) These four graphs demonstrate the performance of the deep learning model across three datasets during the training and validation phases. Each dataset is represented by a unique color (blue for the 1st original image dataset, green for the without background on the image dataset, and red for both original and without background on the image dataset). Metrics such as mAP50-95, mAP50, Precision, and Recall are plotted across 100 epochs to show the learning curve and stability of the model's predictive power over time. (e)-(g) These sets of images are illustrative outcomes from the model's classification task. This display cells labeled as control (CTL) and treated with TGFβ1. Each image exhibits the model's classification with red overlays, where correctly identified regions correspond to the model's prediction. (h)-(j) Shown at the bottom are three confusion matrices corresponding to the model's performance on each of the datasets. Diagonal cells represent correct classifications, whereas off-diagonal cells indicate incorrect predictions, giving a clear quantitative measure of the model's classification accuracy. (For interpretation of the references to color in this figure legend, the reader is referred to the Web version of this article.)

Our model training was conducted over 100 epochs per dataset, with continuous monitoring of performance metrics, including mean average precision (mAP50-95), mAP50, precision, and recall (Fig. 5a–d). The unaltered image dataset (represented by the blue line) demonstrated superior performance across all metrics, achieving a mAP50-95 rate of up to 95%, significantly higher than the 85% and 83% observed in the datasets with the removed background and combined dataset, respectively. This suggests that background elements in the images may enhance the neural network's ability to contextualize and recognize cellular features, aligning with studies indicating that context is crucial for object detection and classification in complex images [40,41].

The efficacy of the post-training classification process was visually represented through overlays on the cellular images, where the network identified and classified the treatment status of the cells (Fig. 5e–g). The confusion matrices provided a clear quantitative measure of the accuracy for each dataset. The matrix for the original dataset showed high accuracy, with true positive rates of 98% for CTL and 99% for TGFβ1-treated samples, highlighting the network's capability in distinguishing between the two categories (Fig. 5h–j).

Validation losses were assessed across several dimensions, including class, box, distribution focus, and segmentation losses (Supplementary Fig. S6-9). The original dataset consistently showed the lowest loss values, with a class loss of 0.249, indicating effective learning from this dataset. Box loss, reflecting the network's ability in localizing objects, was also the lowest for the original dataset at 0.261. However, segmentation losses were higher for the original dataset (1.343) than for the modified datasets, indicating potential challenges in segmenting cellular features against complex backgrounds. This variation in segmentation performance highlights the importance of the dataset composition in training DNNs for biomedical image analysis [42,43].

## 4 Discussion

In this study, we generated alveolar organoids from iPSCs. Recently, organoid-based systems have emerged as useful *in vitro* platforms and have rapidly expanded across various fields of biomedical research. We continuously advanced organoid-based research, applying organoid systems to toxicity screening, disease modeling, and methodological optimization [21,44,45]. Compared with conventional two-dimensional (2D) culture systems, 3D organoids better recapitulate human physiological characteristics, particularly by maintaining alveolar-like architecture that closely resembles the morphology of human lung tissue. Indeed, we previously observed that iPSC-derived AECs exhibited more efficient differentiation in 3D cultures than in 2D monolayers, further supporting the physiological relevance of the organoid system [21]. Despite these advantages, organoid-based systems remain difficult to handle and technically demanding, including increased experimental complexity, higher costs, and considerable effort required for molecular analyses such as RNA or protein extraction. A clear strength of organoid systems is the compatibility with advanced imaging technologies, which enables the rapid and reproducible acquisition of detailed morphological information. Therefore, we integrated organoid-based modeling with AI-driven image analysis in this study.

Our organoids exhibited robust expressions of distal alveolar epithelial markers such as NKX2.1, SFTPC, SFTPB, AGER, and HOPX. Moreover, in our previous work using the same differentiation protocol [45], we successfully generated iPSC-derived AEC2-like cells and confirmed the presence of lamellar bodies, a morphological hallmark of AEC2s. The SFTPC and SFTPB expression levels observed in that study were comparable to those in the present work, further demonstrating the reproducibility and robustness of our differentiation approach. These findings indicate that AOs derived from hiPSCs serve as promising *in vitro* models, reflecting many structural and functional aspects of the distal lung epithelium. However, the complete maturation and full functionality of these cells require further investigation.

To model pulmonary fibrosis (PF), we applied TGFβ1 stimulation to iPSC-derived AOs, which induced morphological and structural alterations closely resembling clinical fibrotic features [46]. The TGFβ1 treatment led to structural and morphological alterations similar to those observed in clinical settings, validating our model as a robust tool for investigating the cellular and molecular dynamics of fibrosis. This validation is crucial for understanding disease mechanisms and developing potential antifibrotic therapeutics [6]. The model's ability to replicate disease-specific scenarios suggests its potential to accelerate the transition from laboratory findings to clinical applications [47]. Specifically, immunofluorescence staining revealed that α-SMA, a marker of myofibroblasts [48], was notably elevated in TGFβ1-treated organoids, indicating fibrotic remodeling within AOs. While this finding suggests that our model successfully recapitulates myofibroblast activation during fibrosis, the precise cellular origin of α-SMA–positive cells in our model remains to be fully elucidated. Given that iPSC-derived alveolar organoids contain both epithelial and mesenchymal populations [44], it is possible that α-SMA expression arises from multiple sources. Some cells may represent pre-existing mesenchymal components that become activated upon TGFβ1 stimulation, while others might originate from epithelial cells undergoing partial EMT or phenotypic remodeling in response to fibrotic cues, as reported in previous studies using alveolar epithelial cells [33,49]. This cellular heterogeneity may reflect the complex epithelial–mesenchymal interactions known to occur in the human alveolar niche during fibrosis [9,50]. Future single-cell–based analyses will be valuable for delineating these distinct cellular trajectories in greater detail.

Moreover, the integration of advanced DNNs for the classification and analysis of the resulting biomedical images has proven pivotal in enhancing the precision of our observations [51]. Sophisticated image processing techniques enabled our DNN to detect and quantify subtle morphological changes induced by TGFβ1, highlighting the transformative potential of AI in medical imaging [40]. The accuracy and precision of our model across different datasets highlight its efficacy and reliability [42]. Notably, our systematic evaluation under various conditions revealed key factors influencing the performance of image classification models, such as dataset quality and complexity of model training [41]. These insights are critical for optimizing DNNs for similar biomedical research. While the current AI-based analysis successfully distinguished TGFβ1-induced fibrotic remodeling from control organoids; however, the generalizability of the model remains limited. As this study represents an early developmental stage of the platform, the algorithm was trained using organoids that exhibit distinct morphological alterations under IPF-like conditions, such as enlargement and structural distortion. However, subtle changes induced by mild external stimuli or epithelial-intrinsic perturbations may not yet be fully captured by the current analytical framework. Future integration of biological information such as fluorescence reporter systems, molecular marker expression, or high-throughput imaging platforms is expected to enhance the model's sensitivity and specificity. Importantly, the high level of accuracy and ability of the model to discern minute differences in organoid morphology supports its application as a valuable tool in the field. This offers a novel approach for researchers to understand disease mechanisms at the granular level and assess the efficacy of new treatments in a controlled, repeatable environment. Our findings advocate for the broader adoption of such models in biomedical research, potentially revolutionizing the study and approach of diseases in preclinical settings.

Although the present study focused primarily on image-derived features to evaluate fibrotic alterations, the model effectively captured biologically meaningful morphological and textural signatures such as increased extracellular matrix density, cellular elongation, and collagen-like fibrillar structures that are consistent with the histopathological hallmarks of fibrosis. These results demonstrate that image-based learning alone can reflect the phenotypic manifestations of TGFβ1-induced fibrogenesis. Nevertheless, it is considered necessary to integrate molecular parameters, including gene expression and protein markers, to further enhance the predictive accuracy and biological interpretability of the model. Such integration would provide a more comprehensive understanding of fibrosis progression and therapeutic response.

In addition, single-cell RNA sequencing (scRNA-seq) would provide an important avenue for further characterizing the cellular composition and heterogeneity of the organoid cultures. While the current system captures key aspects of alveolar epithelial biology and remodeling, additional resolution at the single-cell level would enable a more precise definition of the cellular states present within the cultures. In particular, such approaches may facilitate not only the delineation of distinct cellular trajectories associated with TGFβ1-induced remodeling, but also a more comprehensive assessment of the diversity and relative composition of epithelial cell populations. In this regard, future studies will focus on integrating scRNA-seq analyses with continued refinement of the organoid differentiation protocol to promote more mature and well-defined alveolar phenotypes. These efforts are expected to provide a more detailed and systematic characterization of the cellular landscape within this system, thereby further strengthening its utility for studying dynamic epithelial remodeling processes *in vitro*.

In conclusion, this study demonstrated that TGFβ1-induced alterations in iPSC-derived AOs closely mimic the pathological characteristics of PF, establishing a robust *in vitro* model for elucidating disease mechanisms. The integration of advanced DNNs for image analysis significantly enhanced the identification of subtle morphological changes, confirming the utility of this model for detailed pathological assessments. The precision and accuracy achieved in classifying these changes highlight the potential of combining high-throughput imaging with AI to advance our understanding of fibrotic diseases. This model not only provides novel insights into the cellular and molecular underpinnings of PF but also holds promise for accelerating the development of anti-fibrotic therapies, potentially bridging the gap between preclinical studies and clinical applications. This study establishes a benchmark for leveraging cutting-edge technology to improve disease modeling and therapeutic testing, representing a significant advancement in regenerative medicine and drug discovery.

## CRediT authorship contribution statement

**Seung Hyeon Kim:** Conceptualization, Data curation, Formal analysis, Software, Validation, Visualization, Writing – original draft, Writing – review & editing. **Jooyeon Lee:** Data curation, Formal analysis, Methodology, Validation, Visualization, Writing – original draft, Writing – review & editing. **Jong Hyeok Han:** Data curation, Formal analysis, Methodology, Software, Visualization, Writing – original draft, Writing – review & editing. **Sang Hyuk Seo:** Data curation, Software, Visualization, Writing – original draft, Writing – review & editing. **Jimin Jang:** Writing – review & editing. **Jong-Hee Lee:** Resources, Visualization, Writing – original draft, Writing – review & editing. **Seok-Ho Hong:** Data curation, Supervision, Validation, Writing – original draft, Writing – review & editing. **Myeong Jin Ju:** Data curation, Visualization, Writing – original draft, Writing – review & editing. **Dae-Hee Lee:** Software, Writing – review & editing. **Hee-Jae Jeon:** Conceptualization, Data curation, Formal analysis, Funding acquisition, Investigation, Methodology, Project administration, Software, Supervision, Validation, Visualization, Writing – original draft, Writing – review & editing. **Se-Ran Yang:** Conceptualization, Data curation, Formal analysis, Funding acquisition, Investigation, Methodology, Project administration, Resources, Supervision, Validation, Visualization, Writing – original draft, Writing – review & editing.

## Declaration of competing interest

The authors declare that they have no known competing financial interests or personal relationships that could have appeared to influence the work reported in this paper.

## Data Availability

Data will be made available on request.

## References

[1] Lederer D.J., Martinez F.J. (2018). Idiopathic pulmonary fibrosis. N. Engl. J. Med..

[2] Taskar V., Coultas D. (2008). Exposures and idiopathic lung disease. Semin. Respir. Crit. Care Med..

[3] Jenkins R.G., Moore B.B., Chambers R.C., Eickelberg O., Königshoff M., Kolb M., Laurent G.J., Nanthakumar C.B., Olman M.A., Pardo A. (2017). An official American thoracic society workshop report: use of animal models for the preclinical assessment of potential therapies for pulmonary fibrosis. Am. J. Respir. Cell Mol. Biol..

[4] Stone R.C., Pastar I., Ojeh N., Chen V., Liu S., Garzon K.I., Tomic-Canic M. (2016). Epithelial-mesenchymal transition in tissue repair and fibrosis. Cell Tissue Res..

[5] Wuyts W.A., Agostini C., Antoniou K.M., Bouros D., Chambers R.C., Cottin V., Egan J.J., Lambrecht B.N., Lories R., Parfrey H. (2013). The pathogenesis of pulmonary fibrosis: a moving target. Eur. Respir. J..

[6] Raghu G., Remy-Jardin M., Myers J.L., Richeldi L., Ryerson C.J., Lederer D.J., Behr J., Cottin V., Danoff S.K., Morell F. (2018). Diagnosis of idiopathic pulmonary fibrosis. An official ATS/ERS/JRS/ALAT clinical practice guideline. Am. J. Respir. Crit. Care Med..

[7] Selman M., Pardo A. (2014). Revealing the pathogenic and aging-related mechanisms of the enigmatic idiopathic pulmonary fibrosis: an integral model. Am. J. Respir. Crit. Care Med..

[8] Barkauskas C.E., Cronce M.J., Rackley C.R., Bowie E.J., Keene D.R., Stripp B.R., Randell S.H., Noble P.W., Hogan B.L.M. (2013). Type 2 alveolar cells are stem cells in adult lung. J. Clin. Investig..

[9] Rock J.R., Barkauskas C.E., Cronce M.J., Xue Y., Harris J.R., Liang J., Noble P.W., Hogan B.L.M. (2011). Multiple stromal populations contribute to pulmonary fibrosis without evidence for epithelial to mesenchymal transition. Proc. Natl. Acad. Sci. USA..

[10] Moll S., Ebeling M., Weibel F., Farina A., Del Rosario A.A., Hoflack J.C., Pomposiello S., Prunotto M. (2013). Epithelial cells as active player in fibrosis: findings from an in vitro model. PLoS One.

[11] Jacob A., Vedaie M., Roberts D.A., Thomas D.C., Villacorta-Martin C., Alysandratos K.-D., Hawkins F., Kotton D.N. (2019). Derivation of self-renewing lung alveolar epithelial type II cells from human pluripotent stem cells. Nat. Protoc..

[12] Kotton D.N., Morrisey E.E. (2014). Lung regeneration: mechanisms, applications and emerging stem cell populations. Nat. Med..

[13] Clevers H. (2016). Modeling development and disease with organoids. Cell.

[14] Sachs N., De Ligt J., Kopper O., Gogola E., Bounova G., Weeber F., Balgobind A.V., Wind K., Gracanin A., Begthel H. (2018). A living biobank of breast cancer organoids captures disease heterogeneity. Cell.

[15] Barker N. (2014). Adult intestinal stem cells: critical drivers of epithelial homeostasis and regeneration. Nat. Rev. Mol. Cell Biol..

[16] Marioni J.C., Arendt D. (2017). How single-cell genomics is changing evolutionary and developmental biology. Annu. Rev. Cell Dev. Biol..

[17] Takebe T., Wells J.M. (2019). Organoids by design. Science.

[18] Turner D.A., Baillie-Johnson P., Martinez Arias A. (2016). Organoids and the genetically encoded self-assembly of embryonic stem cells. Bioessays.

[19] Wynn T.A. (2008). Cellular and molecular mechanisms of fibrosis. J. Pathol..

[20] Hofer M., Lutolf M.P. (2021). Engineering organoids. Nat. Rev. Mater..

[21] Lee J., Baek H., Jang J., Park J., Cha S.-R., Hong S.-H., Kim J., Lee J.-H., Hong I.-S., Wang S.-J. (2023). Establishment of a human induced pluripotent stem cell derived alveolar organoid for toxicity assessment. Toxicol. Vitro.

[22] Jeon H.-J., Leem J.W., Ji Y., Park S.M., Park J., Kim K.-Y., Kim S.-W., Kim Y.L. (2022). Cyber-physical watermarking with inkjet edible bioprinting. Adv. Funct. Mater..

[23] Leem J.W., Jeon H.-J., Ji Y., Park S.M., Kwak Y., Park J., Kim K.-Y., Kim S.-W., Kim Y.L. (2022). Edible matrix code with photogenic silk proteins. ACS Cent. Sci..

[24] B. Selcuk, T. Serif, in: International Conference on Mobile Web and Intelligent Information Systems, Springer, 161–174.

[25] Heo B., Chun S., Oh S.J., Han D., Yun S., Kim G., Uh Y., Ha J.-W. (2020). ADAMP: slowing down the slowdown for momentum optimizers on scale-invariant weights. arXiv preprint arXiv:2006.08217.

[26] Wang X., Song J. (2021). ICIoU: improved loss based on complete intersection over union for bounding box regression. IEEE Access.

[27] Pärnamaa T., Parts L. (2017). Accurate classification of protein subcellular localization from high-throughput microscopy images using deep learning. G3 (Bethesda).

[28] Rawat W., Wang Z. (2017). Deep convolutional neural networks for image classification: a comprehensive review. Neural Comput..

[29] Raghavendra S., Rao D., Abhilash S.K., Nookala V.M., Bharathi P.G. (2023). Elevating amodal segmentation using ASH-net architecture for accurate object boundary estimation. IEEE Access.

[30] Gotoh S., Ito I., Nagasaki T., Yamamoto Y., Konishi S., Korogi Y., Matsumoto H., Muro S., Hirai T., Funato M. (2014). Generation of alveolar epithelial spheroids via isolated progenitor cells from human pluripotent stem cells. Stem Cell Rep..

[31] Wang Y., Tang Z., Huang H., Li J., Wang Z., Yu Y., Zhang C., Li J., Dai H., Wang F. (2018). Pulmonary alveolar type I cell population consists of two distinct subtypes that differ in cell fate. Proc. Natl. Acad. Sci. USA.

[32] Jain K.G., Liu Y., Zhao R., Muire P.J., Zhang J., Zang Q.S., Ji H.-L. (2024). Humanized L184Q mutated surfactant protein c gene alters alveolar type 2 epithelial cell fate. Int. J. Mol. Sci..

[33] Willis B.C., Liebler J.M., Luby-Phelps K., Nicholson A.G., Crandall E.D., Du Bois R.M., Borok Z. (2005). Induction of epithelial-mesenchymal transition in alveolar epithelial cells by transforming growth factor-β1: potential role in idiopathic pulmonary fibrosis. Am. J. Pathol..

[34] Saito A., Horie M., Nagase T. (2018). TGF-β signaling in lung health and disease. Int. J. Mol. Sci..

[35] Mauviel A. (2005). Fibrosis Research: Methods and Protocols.

[36] Leask A., Abraham D.J. (2004). TGF-β signaling and the fibrotic response. FASEB J..

[37] Cook D.P., Vanderhyden B.C. (2020). Context specificity of the EMT transcriptional response. Nat. Commun..

[38] Hinz B., Phan S.H., Thannickal V.J., Galli A., Bochaton-Piallat M.-L., Gabbiani G. (2007). The myofibroblast: one function, multiple origins. Am. J. Pathol..

[39] Wilson M.S., Wynn T.A. (2009). Pulmonary fibrosis: pathogenesis, etiology and regulation. Mucosal Immunol..

[40] Redmon J., Divvala S., Girshick R., Farhadi A. (2009). Proceedings of the IEEE Conference on Computer Vision and Pattern Recognition.

[41] Lin T.-Y., Goyal P., Girshick R., He K., Dollár P. (2017). Proceedings of the IEEE International Conference on Computer Vision.

[42] He K., Gkioxari G., Dollár P., Girshick R. (2017). Proceedings of the IEEE International Conference on Computer Vision.

[43] Long J., Shelhamer E., Darrell T. (2015). Proceedings of the IEEE Conference on Computer Vision and Pattern Recognition.

[44] Kim J.-H., An G.H., Kim J.-Y., Rasaei R., Kim W.J., Jin X., Woo D.-H., Han C., Yang S.-R., Kim J.-H. (2021). Human pluripotent stem cell-derived alveolar organoids for modeling pulmonary fibrosis and drug testing. Cell Death Discov..

[45] Heo H.-R., Kim J., Kim W.J., Yang S.-R., Han S.-S., Lee S.J., Hong Y., Hong S.-H. (2019). Human pluripotent stem cell-derived alveolar epithelial cells are alternatives for in vitro pulmotoxicity assessment. Sci. Rep..

[46] Selman M., Pardo A., Barrera L., Estrada A., Watson S.R., Wilson K., Aziz N., Kaminski N., Zlotnik A. (2006). Gene expression profiles distinguish idiopathic pulmonary fibrosis from hypersensitivity pneumonitis. Am. J. Respir. Crit. Care Med..

[47] Wolters P.J., Collard H.R., Jones K.D. (2014). Pathogenesis of idiopathic pulmonary fibrosis. Annu. Rev. Pathol..

[48] Hung C.F. (2020). Origin of myofibroblasts in lung fibrosis, Curr. Tissue Microenviron. Rep.

[49] Marmai C., Sutherland R.E., Kim K.K., Dolganov G.M., Fang X., Kim S.S., Jiang S., Golden J.A., Hoopes C.W., Matthay M.A. (2011). Alveolar epithelial cells express mesenchymal proteins in patients with idiopathic pulmonary fibrosis. Am. J. Physiol. Lung Cell. Mol. Physiol..

[50] Habermann A.C., Gutierrez A.J., Bui L.T., Yahn S.L., Winters N.I., Calvi C.L., Peter L., Chung M.-I., Taylor C.J., Jetter C. (2020). Single-cell RNA sequencing reveals profibrotic roles of distinct epithelial and mesenchymal lineages in pulmonary fibrosis. Sci. Adv..

[51] Strieter R.M., Mehrad B. (2009). New mechanisms of pulmonary fibrosis. Chest.

