# Supplementary material for: AI-enabled label free monitoring of TGFβ1-induced remodeling in iPSC-derived alveolar organoids
Source: Mater Today Bio. 2026 May 7;38:103181. doi: 10.1016/j.mtbio.2026.103181 (PMC13224076; doi:10.1016/j.mtbio.2026.103181)
Supplement: Multimedia component 1 [file mmc1.docx]

SUPPLEMENTARY MATERIALS

Fig S1 to S9

**
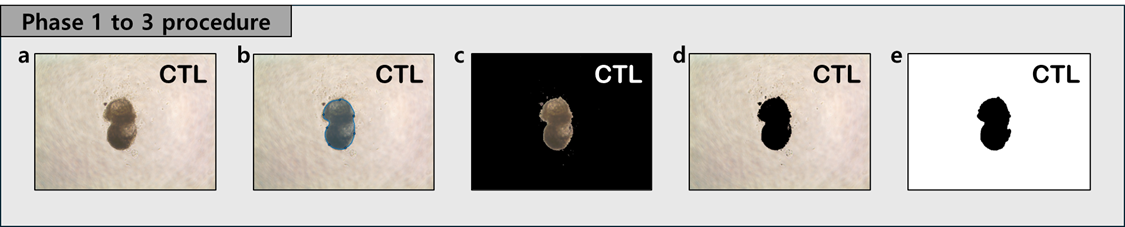
**

**Fig. S1.** Detailed images processing steps for automatic ROI tracking and background subtraction. (a). Finding the alveolar organoid. (b). Select the region of alveolar organoid for ROI. (c). Extracting the ROI (Phase e) (d). Separate the extracted ROI and background. (e). Save the ROI for labeling. (Phase e)

**
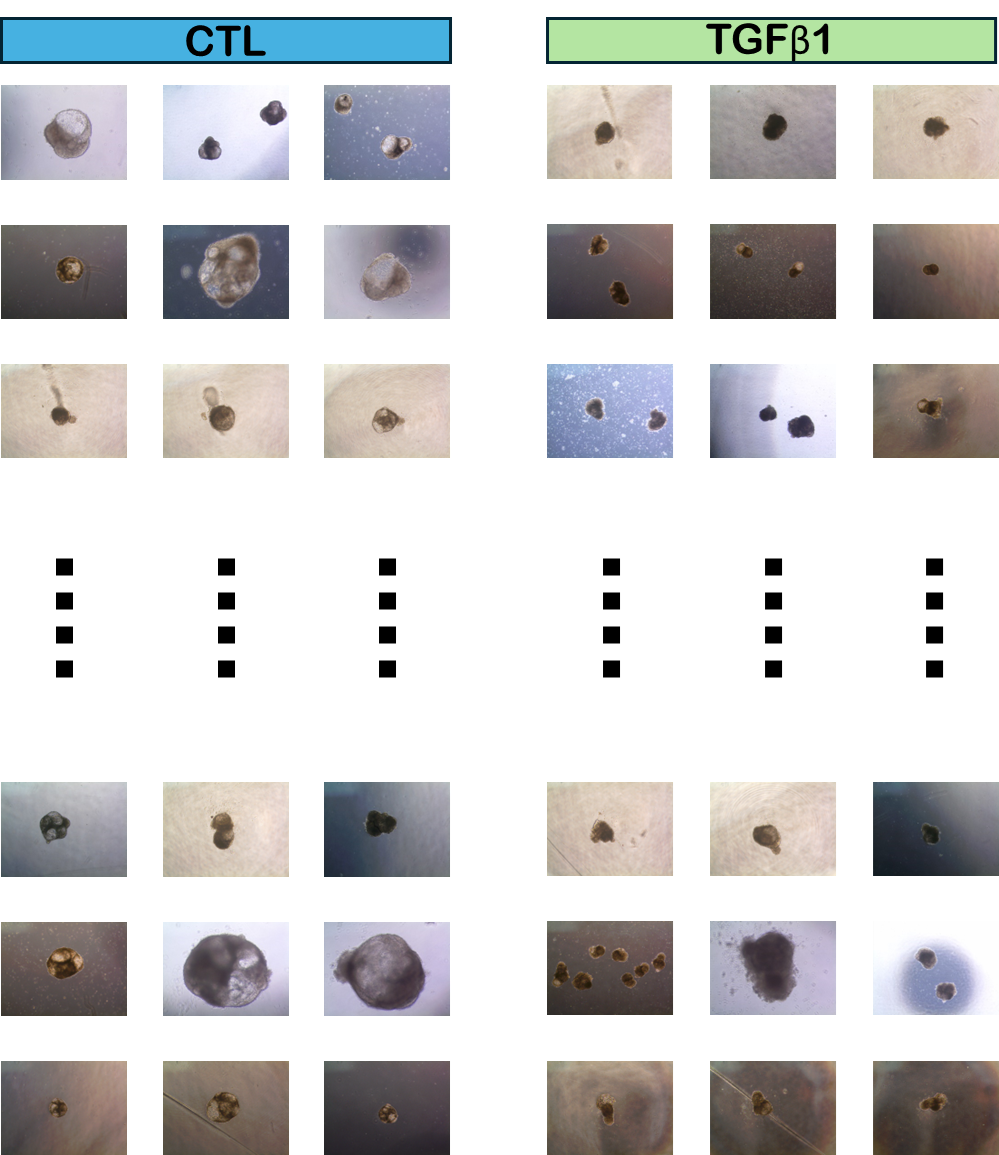
**

**Fig. S2.** Total images of alveolar organoids between control group and treated TGFβ1.


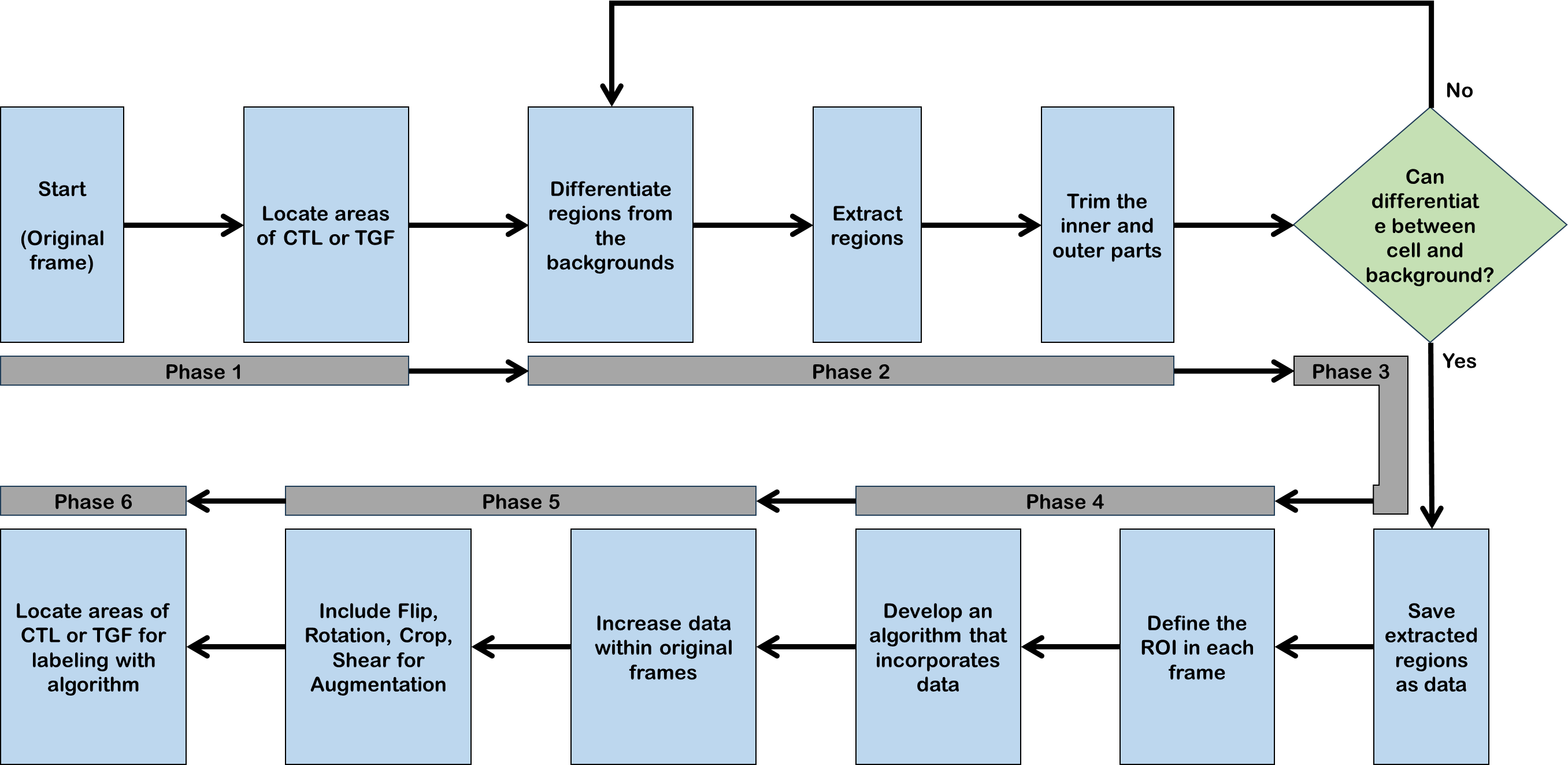


**Fig. S3.** Flowchart showing the algorithm for the processing images of the alveolar organoids.

**
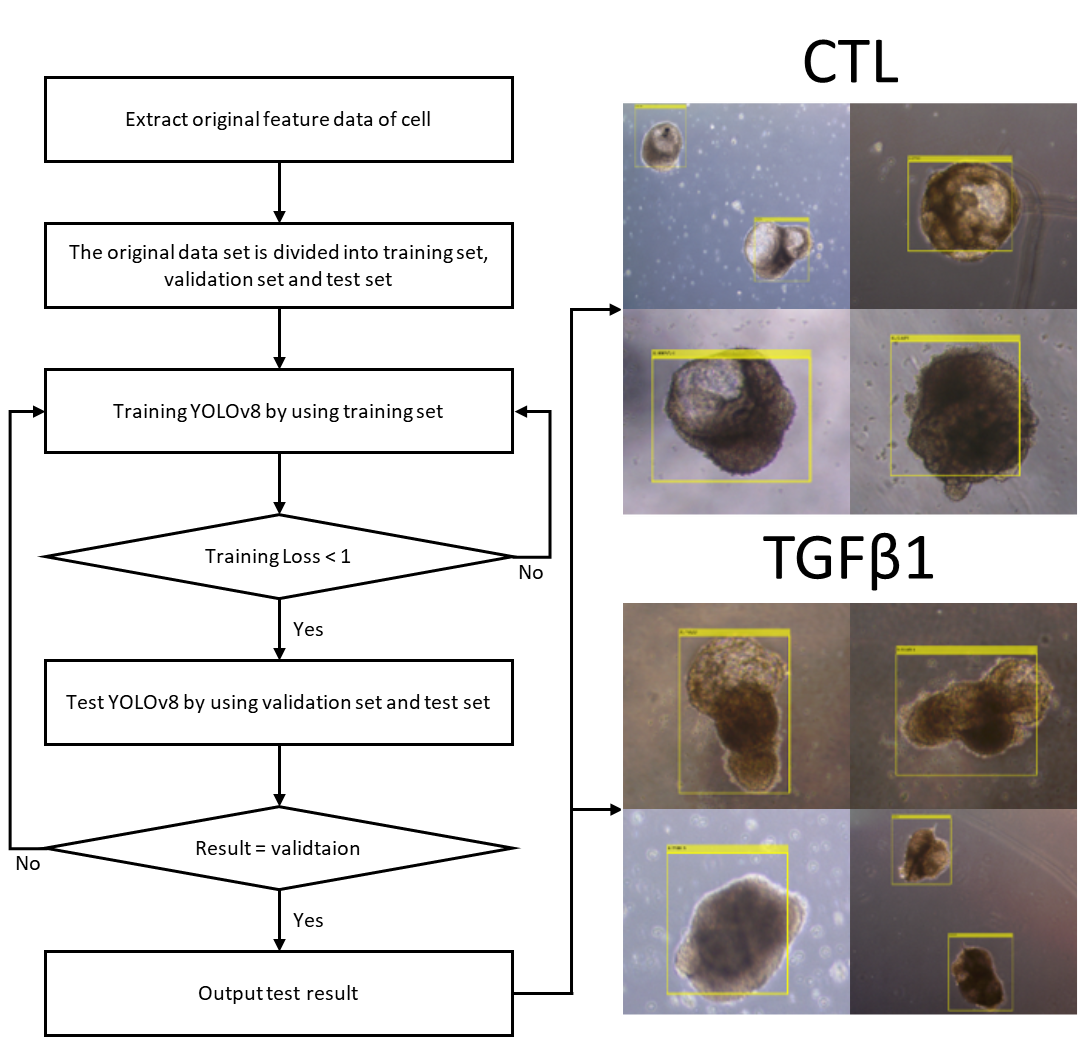
**

**Fig. S4.** Algorithm-driven progression analysis in alveolar organoid classification.


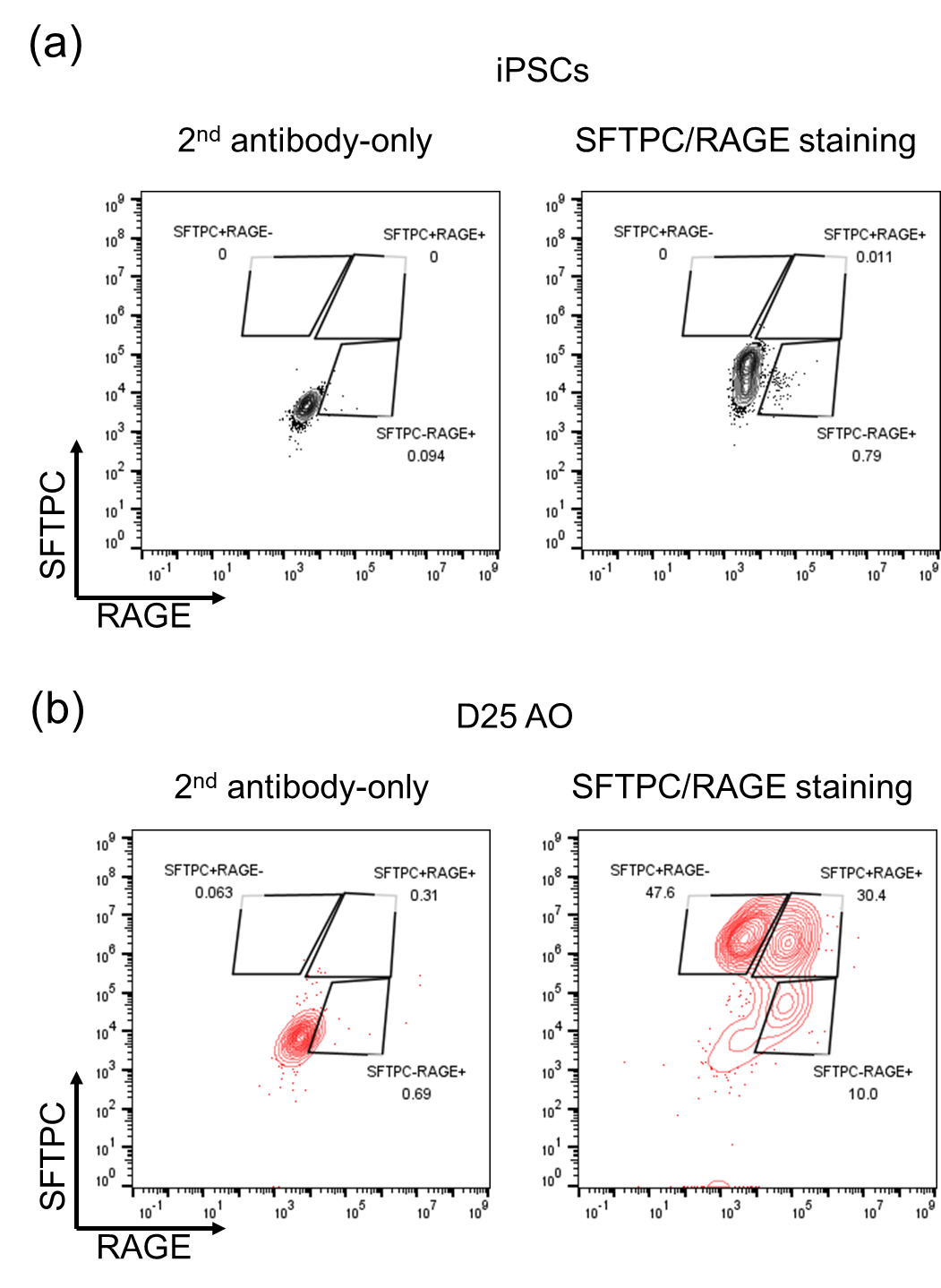


**Fig. S5. Flow cytometric analysis of SFTPC and RAGE expression in iPSCs and alveolar organoids.** (a) Representative flow cytometry plots showing SFTPC and RAGE expression in undifferentiated iPSCs, with the secondary-only control (left) and SFTPC/RAGE staining (right). (b) Representative flow cytometry plots showing SFTPC and RAGE expression in AOs at day 25, with the secondary-only control (left) and SFTPC/RAGE staining (right).

**
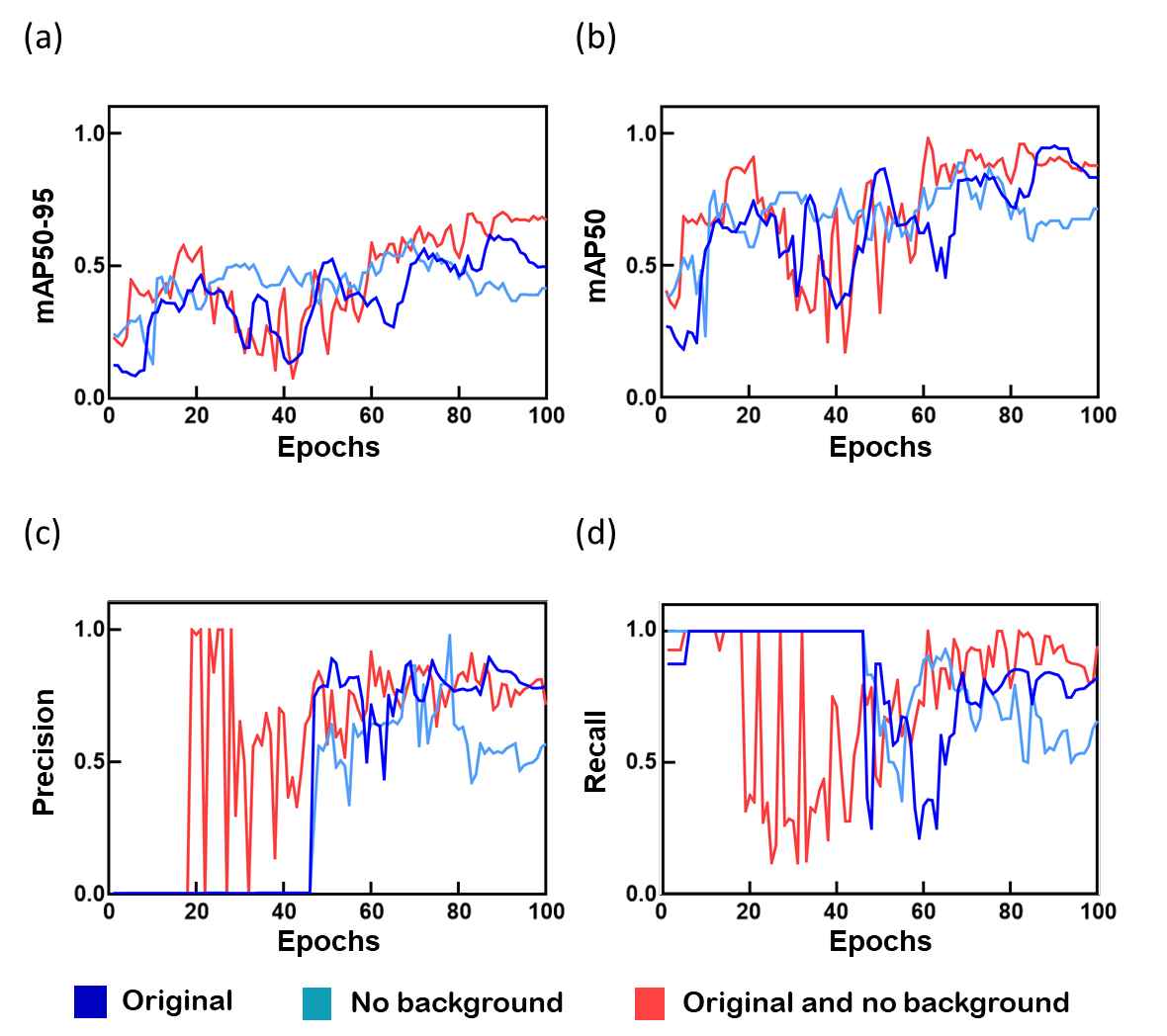
**

**Fig. S6.** Alveolar organoid imaging spectral comparison and evaluation over the epochs with original dataset. (a) Estimation of mAP50-95 with alveolar organoid spectral profile variances under different background conditions. (b) Background noise influence on alveolar organoid spectral data with mAP50. (c) The precision of classifying alveolar organoid by model. (d) The recall signal patterns show the ratio of the model has classified alveolar organoid among all the objects.

**
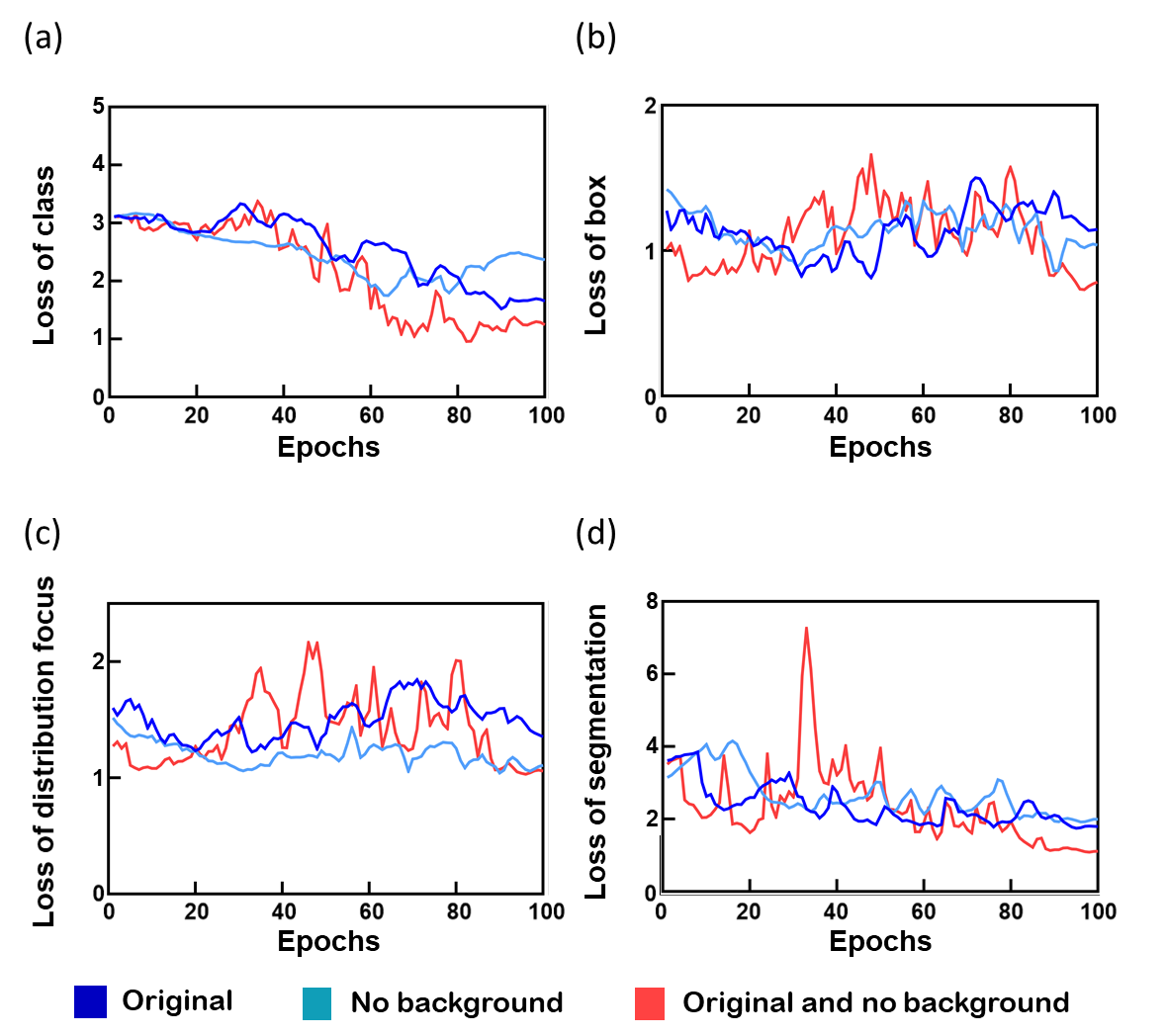
**

**Fig. S7.** Spectral analysis of alveolar organoid imaging and background influence with original dataset. (a) Loss of class of spectral profile. (b) Loss of box Organoid spectral profile. (c) Loss of distribution focus of spectral data for even training. (d) Loss of segmentation results.


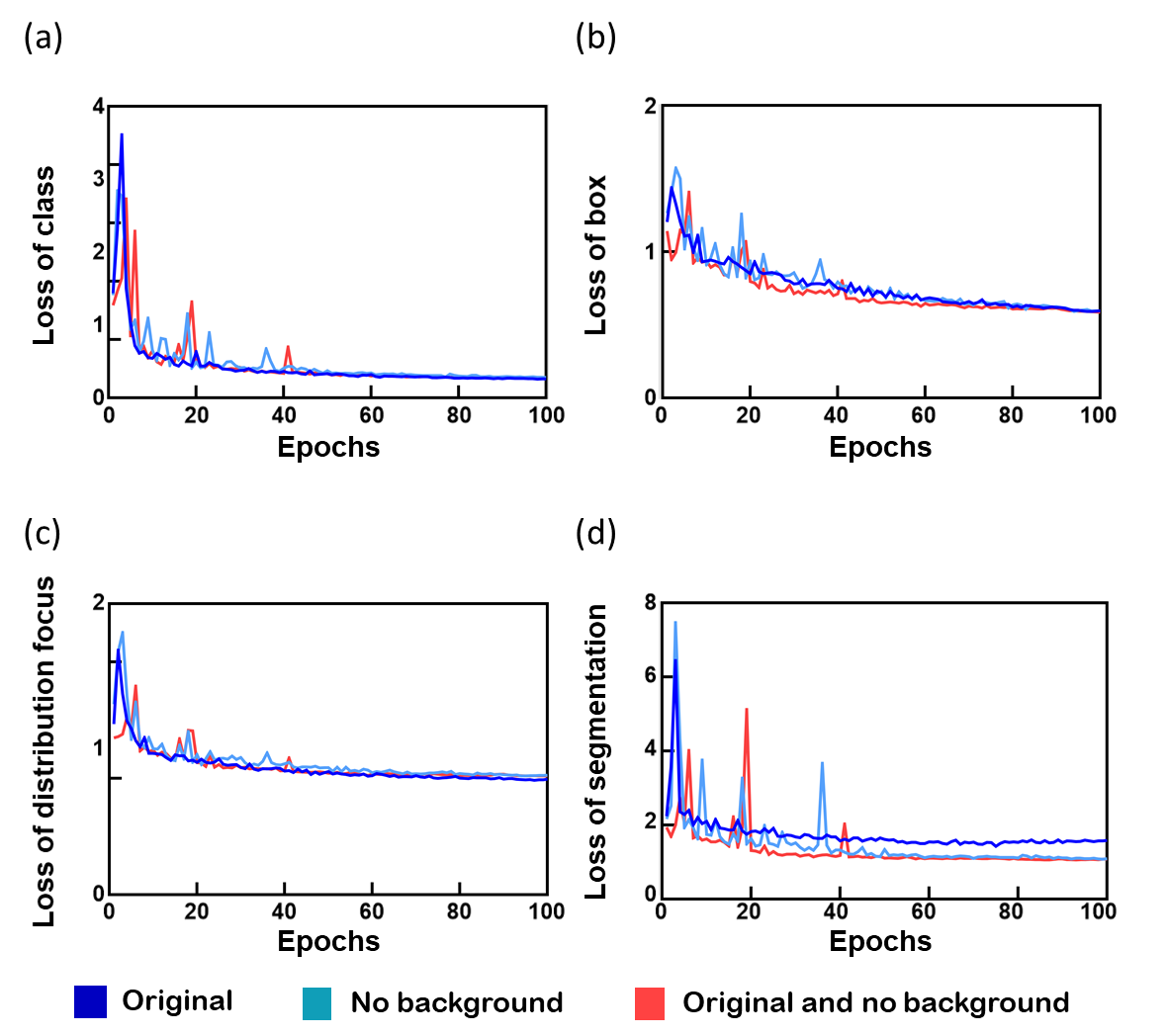


**Fig. S8.** Comparative spectral analysis of alveolar organoid imaging and the effect of background on signal detection with augmented dataset. (a) Loss of class of spectral profile. (b) Loss of box Organoid spectral profile. (c) Loss of distribution focus of spectral data for even training. (d) Loss of segmentation results.


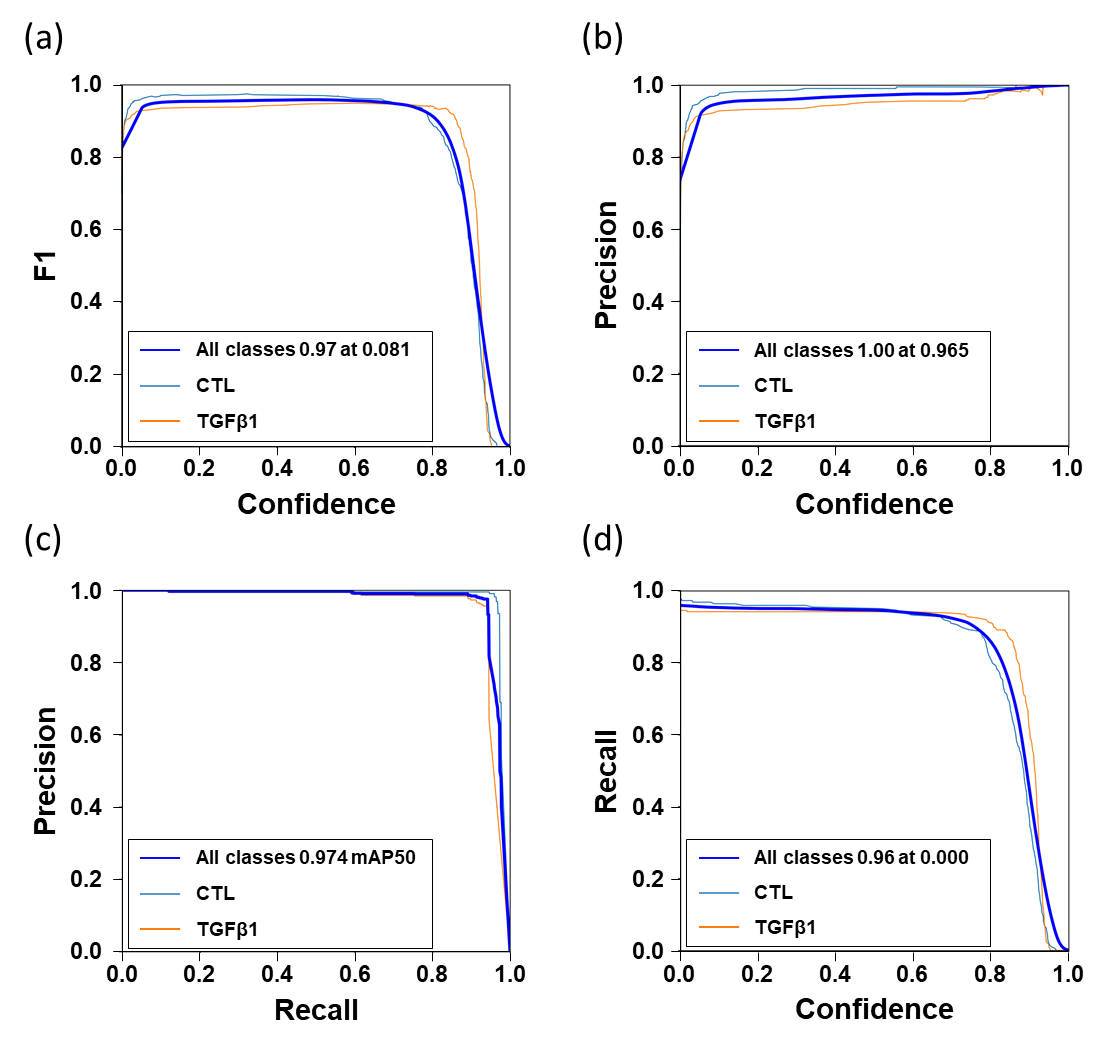


**Fig. S9.** The results of experiment that used original dataset with augmented dataset.

(a) The F1 score shows a balanced metric that precision and recall. (b) The precision-confidence curve shows the ratio of correctly detected objects to the total number of objects detected by the model. (c) The precision-recall curve is a graph that plots precision against recall. (d) Recall-confidence curve that reflects the confidence threshold.
